# Effects of Quercetin on CYP450 and Cytokines in Aroclor 1254 Injured Endometrial Cells of the Pregnant Rats

**DOI:** 10.1155/2014/497508

**Published:** 2014-03-10

**Authors:** Lina Xu, Liyun Sun, Liqin Lu, Xiuhui Zhong, Yuzhong Ma, Jianhua Qin

**Affiliations:** ^1^College of Veterinary Medicine, Agricultural University of Hebei, Baoding, Hebei 071001, China; ^2^Institute of Traditional Chinese Veterinary Medicine, Agricultural University of Hebei, Baoding, Hebei 071001, China

## Abstract

Polychlorinated biphenyls (PCBs) are widespread persistent residual environmental pollutants, which affect seriously the growth and reproductive alterations in humans and animals. Aroclor 1254 is a commercial mixture of PCBs. Quercetin is a flavonoid, which acts on estrogen receptors and causes the development of estrogen-related diseases. In this paper, the primary cultured endometrial cells in the pregnant rats were isolated and Aroclor 1254 was used to induce the injured endometrial cells model. The cells were treated with gradient quercetin, the viability of the endometrial cells, the expressions of CYP450, the contents of TNF-**α**, IL-6, estradiol (E_2_), and progesterone (P_4_) were measured. It showed that the viability of the cultured endometrial cells, the expression of CYP1A1 and CYP2B1, and the contents of TNF-**α**, E_2_, and IL-6 in the injured endometrial cells increased with the treatment of quercetin. It shows that quercetin has protective effect on the injured endometrial cells in the pregnant rats, this provide a basis on herbal medicine protection for animal reproductive diseases caused by environmental endocrine disruptors.

## 1. Introduction

Polychlorinated biphenyls (PCBs) are widespread persistent residual environmental pollutants, which have been widely used for various industrial applications [1]. PCBs affect seriously the growth and reproductive alterations in humans and animals [2–4]. PCBs convert into hydroxy-PCBs in the liver [5, 6]; hydroxy-PCBs produced estrogen and thyroid interference effects in the body and caused serious influence to reproductive functions [7]. PCB can result in an imbalance in the cellular oxidative stress/antioxidant status and thus cause cell injury; oxidative stress can play a critical role in observed PCB mediated endothelial cell dysfunction. Higher dosages of PCBs adversely affect fertilization and cause degeneration of oocytes and abnormality in the early mouse embryo [8]. The sperm capacitation, fertilized egg implantation, and fetal development occur in mammal uterus. Crinnion [9] and Fadhel et al. [10] found that most of the PCBs congeners could induce the metabolic enzymes* in vivo* through the aryl hydrocarbon (Ah) receptor signal transduction pathway.

Quercetin is a flavonoid, which is found in vegetables, fruits, and other dietary sources [11]. It is marketed as a diet supplement with anti-inflammatory, antiviral, immunomodulatory, and antioxidant properties [12, 13]. Quercetin is a scavenger of O_2_–, NO–, HO–, and peroxy radicals. In addition, previous studies have shown that quercetin inhibited the oxidative DNA damage induced by hydrogen peroxide [14]. The chemical structure of quercetin is similar to the mammal estrogen. As an estrogen receptor regulator, quercetin has high affinity to *α*-ligand binding domain of estrogen receptor [15, 16]. Quercetin acts on estrogen receptors and causes the occurrence and development of estrogen-related diseases.

Several studies suggest that the oxidative stress induced by specific environmental contaminants, that is, aromatic hydrocarbons like PCB 77, is due to the interaction of these compounds with the aryl hydrocarbon receptor (AhR) [1]. CYP1A1 belongs to the hormone metabolism enzymes. The CYP2B associates with xenobiotic detoxification [17, 18]. Indeed, PCB mixtures or individual congeners are effective mixed function oxidase system inducers. As a commercial mixture of PCBs, Aroclor 1254 impacts the growth and development of uterus in different stages of female rats and causes the damage of the normal uterine tissue structures. In this paper, the endometrial cells in the pregnant rats are isolated and cultured, the injured endometrial cells model is prepared by Aroclor 1254 induction, the model cells are treated with quercetin, and the expressions of CYP450 are determined by RT-PCR and Western blot so as to study whether quercetin has protective effect on the injured endometrial cells in the pregnant rats, to provide a basis on herbal medicine protection for animal reproductive diseases caused by environmental endocrine disruptors.

## 2. Materials and Methods

### 2.1. Chemicals and Reagents

Aroclor 1254 was purchased from AccuStandard, Inc., New Haven, USA. Quercetin was purchased from Sigma-Aldrich Co., Louis, USA. CYP1A1 and CYP2B1 antibodies were purchased from Chemicon, USA. DMEM/F-12 medium and Trizol were purchased from Invitrogen, USA. Fetal bovine serum (FBS) was from HyClone, Logan, UT. NBT, BCIP were purchased from Amresco, USA. M-MLV reverse transcriptase was purchased from Promega, USA; RT-PCR primers were synthetized by Sangon Biotech Co. Ltd, Shanghai, China. TNF-*α*, IL-6, E_2_, and P_4_ ELISA kits were purchased from Biovalue, Shanghai, China.

### 2.2. Experimental Animals

Ten-week-old naive female and male Sprague-Dawley rats were purchased from the Experimental Animal Center of Hebei Medical University, China. They were housed in polypropylene cages and maintained under standard laboratory conditions with a 12 h light-dark cycle and free access to standard rat pellet diet and drinking water. They were acclimatized to laboratory conditions for 10 days before starting the experiment. The weight of the female rats was 180–220 g and of the male rats was 250–300 g. Pregnancies were obtained by housing one estrous female with one male overnight, and the females were examined each day in the early morning for the presence of sperm via vaginal smear. The detection day of the sperm was designated as day 0 of pregnancy.

### 2.3. Isolation and Culture of Endometrial Cells

On day 5.5 of gestation, the rat was sacrificed by cervical dislocation.The uterus of the pregnant Sprague-Dawley rat was rinsed with D-Hanks solution. The endometrial cells were scraped and digested by 0.25% Trypsin-EDTA at 37°C water bath for 5–10 min. The filtered solution was centrifuged at 1200 rpm for 10 min, and the centrifugation was repeated for 3 times. The pellet was suspended in DMEM/F-12 medium containing 15% FBS, and the viability was about 95% determined by Trypan blue exclusion. The endometrial cells were then inoculated into culture plate at a density of 6 × 10^5^/per well (12-well plate) and cultured at 37°C/5% CO_2_ until the cells were confluent.

### 2.4. Establishment of the Injured Endometrial Cells Model Induced by Aroclor 1254

The density of 1 × 10^5^/well endometrial cells in 96-well plate was treated with gradient concentrations of Aroclor 1254 and incubated at 37°C/5% CO_2_ for 48 h. The cells were incubated by the treatment of 5 mg/mL MTT 20 *μ*L for 6 h. Then add DMSO 100 *μ*L and vortex for 5 min. The viability of the endometrial cells was measured, and the configuration of endometrial cells was observed. The optimal Aroclor 1254 concentration which impacted the endometrial cells was determined to make the injured endometrial cells model.

### 2.5. Treatment of the Injured Endometrial Cells with Quercetin

The quercetin was dissolved with DMEM/F-12 medium and filtered with 0.22 *μ*m microfilter. The injured rat endometrial cells were treated with gradient concentrations of quercetin for 24–72 h, respectively. The viability of the endometrial cells was measured by MTT method.

### 2.6. CYP450 Expressions by RT-PCR Analysis

The total RNA of the injured endometrial cells after quercetin treatment was obtained by Trizol and quantified. Total RNA (5 *μ*g) was subjected to the synthesis of the first-strand cDNA with random primers and M-MLV reverse transcriptase. The cDNA was subjected to PCR amplification (total volume 50 *μ*L). The cycling parameters were 94°C for 30 s, Tm-5°C for 30 s, and 72°C for 1 min for a total of 30 cycles. The primers for CYP1A1 amplification were 5′-CTGGTTCTGGATACCCAGCTG-3′ (forward) and 5′-CCTAGGGTTGGTTACCAGG-3′ (reverse). The primers for CYP2B1 amplification were 5′-TATCTTGCTCCTCCTTGCTCT-3′ (forward) and 5′-GCCTCCTTTATGGTGTCTGTC-3′ (reverse), and the primers for *β*-actin amplification were 5′-CTTCGACATCACGGCTGATGG-3′ (forward) and 5′-CAGGACCTGTATGCTTCAGG-3′ (reverse). The volume of RT-PCR reaction was 50 *μ*L (2X Taq PCR Master Mix 25 *μ*L, 10 *μ*M forward primer 2 *μ*L, 10 *μ*M reverse primer 2 *μ*L, template 5 *μ*L, and water 16 *μ*L). The PCR was performed as follows: 30 cycles of template denaturation at 94°C for 5 min, primer annealing at Tm-5°C for 30 s, and primer extension at 72°C for 1 min, followed by a final extension at 72°C for 5 min. The PCR-amplified products were resolved by 2% agarose gel electrophoresis.

### 2.7. Western Blot Analysis of CYP450 Expressions

The proteins were obtained by cell lysis buffer, quantified and denatured, run in 12% SDS-PAGE, and transferred into nitrocellulose membranes. The nitrocellulose membranes were blocked with 5% nonfat milk and incubated with CYP450 antibodies. Subsequently, IgG conjugated with alkaline phosphatase was added. The expression of CYP450 was detected by NBT/BCIP. The relative intensities were quantified by KODAK 1D Image Analysis Software.

### 2.8. ELISA Measurement of TNF-*α*, IL-6, E_2_, and P_4_

The contents of TNF-*α*, IL-6, E_2_, and P_4_ in the control group, the injured endometrial cells model group, and the quercetin treating group were measured by the ELISA, respectively, according to the manufacturer's instructions.

### 2.9. Statistical Analysis

Protein concentrations were determined with BCA assay (Pierce) with albumin as standard. Data are presented as mean ± S.E.M. of at least three separate experiments, except where results of blots are shown, in which case a representative experiment is depicted in the figures. The data of absorbance value and ELISA were expressed as mean ± S.E.M. SPSS 13.0 was used to analyze the data, significant differences were compared among groups by one-way analysis of variance (ANOVA), and *P* < 0.05 was considered statistically significant.

## 3. Results

### 3.1. Rat Endometrial Cells Configuration

**Figure 1:**
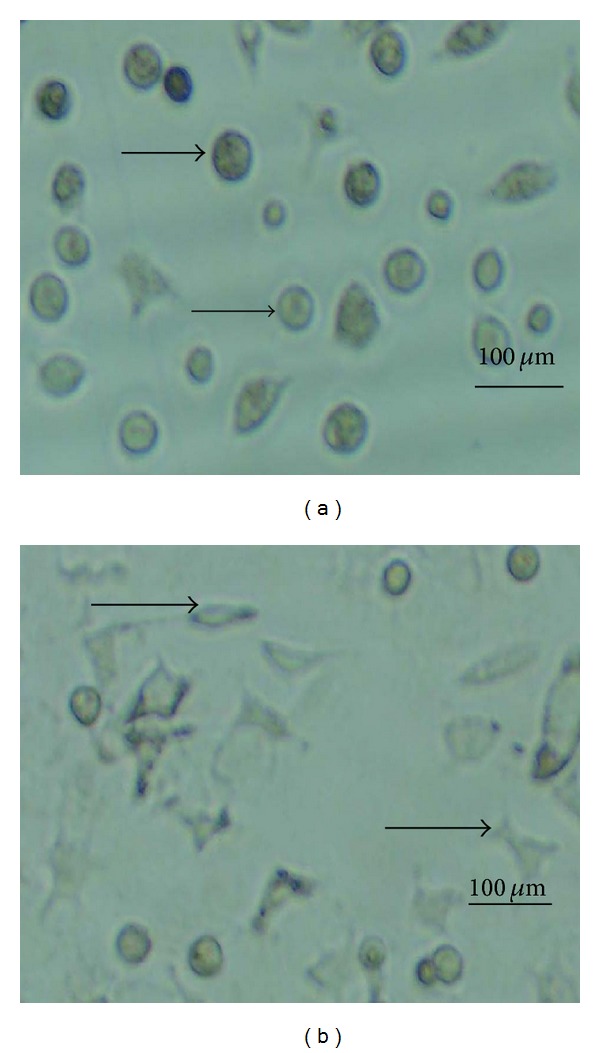
Representative photographs of rat endometrial cells morphological analysis. (a) The rat endometrial cells were incubated for 24 h; most of cells were globular as the arrow indicated. (b) The rat endometrial cells were incubated for 48 h, and their morphologies were spindle or polygonal.

By observation under inverted microscope, the rat endometrial cells grew well. After 24 h incubation, most of cells were globular (Figure 1(a)). With 48 h incubation, the cells showed spindly or polygonal (Figure 1(b)).

### 3.2. Selection of the Optimal Concentration of Aroclor 1254 for the Endometrial Cells Injured Model

**Figure 2:**
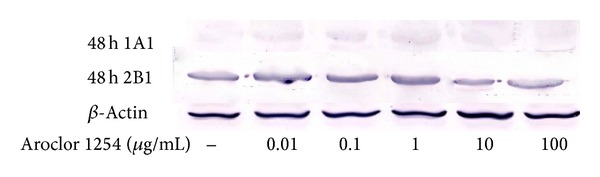
With Aroclor 1254 treatment for 48 h, the CYP1A1 and CYP2B1 expressions in cells were measured by Western blot analysis.

**Figure 3:**
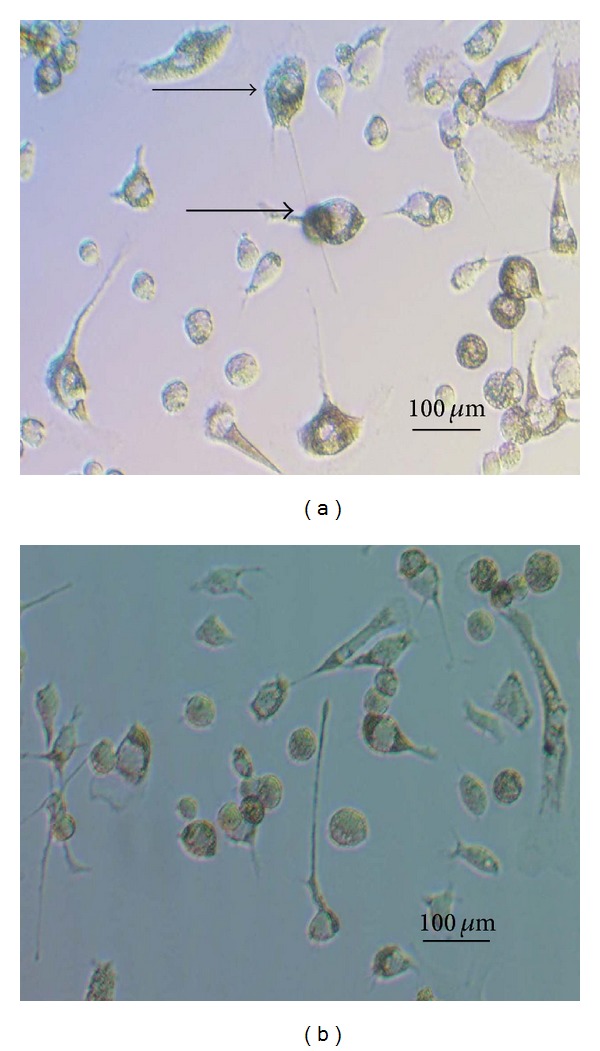
Representative photographs of rat endometrial cells morphological analysis. (a) The rat endometrial cells were treated with 10 *μ*g/mL Aroclor 1254 for 48 h, as the arrow indicated, the endometrial cells looked shrank, some cells were swollen or showed less cytoplasm, even the vacuoles appeared inside the endometrial cells. (b) The injured rat endometrial cells were treated with 50 *μ*mol/L quercetin for 24 h; the configuration of injured endometrial cells turned to normal.

The inhibition ratio (IR) increased with the gradient concentration of Aroclor 1254 treatment. It is shown that nearest IR = 10 was 10 *μ*g/mL Aroclor 1254 (Table 1). The expression of CYP1A1 and CYP2B1 increased as the Aroclor 1254 dose increased, the highest amount of CYP1A1 and CYP2B1 expression was in 1 *μ*g/mL group, and the expression decreased with treatment 10 *μ*g/mL Aroclor 1254 group (Figure 2). In Figure 3(a), the endometrial cells shrank, some cells swelled or showed less cytoplasm, even the vacuoles appeared inside the endometrial cells. Therefore, 10 *μ*g/mL Aroclor 1254 was the optimal concentration for inducing the endometrial cells injured model.

### 3.3. The Optimal Concentration Selection of Quercetin

With the gradient concentration of quercetin treatment, the viability of the cultured endometrial cells increased. The optimal viability of cultured endometrial cells was 50 *μ*mol/L quercetin treated for 24 h (Table 2). The configuration of injured endometrial cells in the model group turned to normal with the treatment of 50 *μ*mol/L quercetin. Therefore, 50 *μ*mol/L quercetin was the optimal concentration for the injured endometrial cells protection (Figure 3(b)).

### 3.4. The Expression of CYP1A1 in Rat Injured Endometrial Cells Treated with Quercetin

**Figure 4:**
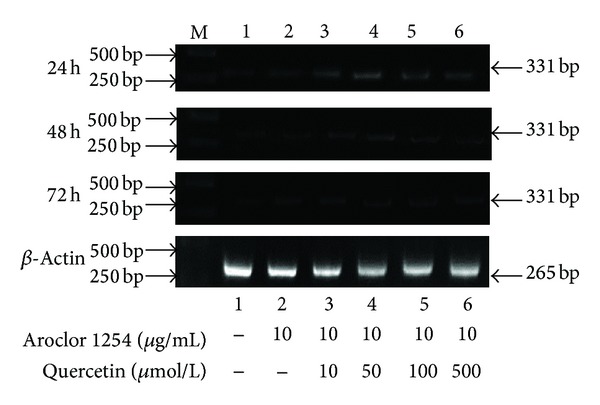
The injured endometrial cells were treated with gradient quercetin for 24 h, 48 h, and 72 h; the CYP1A1 levels in cells were measured by RT-PCR analysis.

From the results of RT-PCR, we can see that the expression of CYP1A1 in the injured endometrial cells increased gradually with the treatment of the gradient quercetin for 24 h; the highest amount of CYP1A1 expression was in 50 *μ*mol/L quercetin group (Figure 4). But there was no CYP1A1 expression by Western blot analysis.

### 3.5. The Expression of CYP2B1 in Rat Injured Endometrial Cells Treated with Quercetin

**Figure 5:**
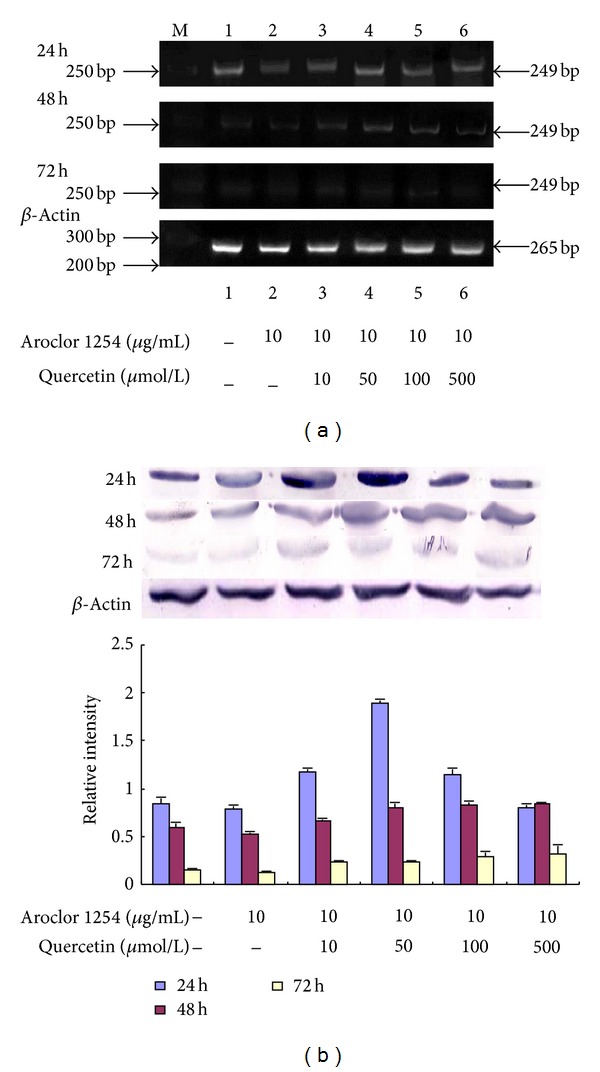
Quercetin induction of CYP2B1 expression in the injured endometrial cells by RT-PCR and Western blot analysis. (a) The CYP2B1 levels in cells were measured by RT-PCR analysis.(b) The CYP2B1 levels in cells were measured by Western blot analysis.

The injured rat endometrial cells were treated with various concentrations of quercetin preparation for 24 h, 48 h, and 72 h, respectively. By RT-PCR and Western Blot methods, it showed that the expression of CYP2B1 increased gradually with the treatment of the gradient quercetin, and the highest amount of CYP2B1 expression was with 50 *μ*mol/L quercetin treated for 24 h (Figure 5).

### 3.6. Effect of 50 *μ*mol/L Quercetin on the Contents of TNF-*α*, IL-6, E_2_, and P_4_ in Injured Endometrial Cells

The contents of TNF-*α*, IL-6, and E_2_ in the injured endometrial cells of the Aroclor 1254 group increased significantly than those in the control group (*P* < 0.05). P_4_ decreased significantly than those in the control group (*P* < 0.05). In the 50 *μ*mol/L quercetin treatment group, the contents of TNF-*α*, IL-6, and E_2_ decreased significantly (*P* < 0.05); P_4_ increased significantly (*P* < 0.05) when compared with the Aroclor 1254 group. There was no significant difference of the contents of IL-6, E_2_, and P_4_ between the quercetin group and the control group (*P* > 0.05) (Table 3).

## 4. Discussion

As the stable physicochemical characteristics and low degradability PCBs are highly accumulative and toxic and usually cause serious damages to the environment, health of human beings and animals. Aroclor 1254 is the typical commercial mixture of PCBs. As environmental endocrine regulator, Aroclor 1254 directly or indirectly interferes with female reproductive functions, impairs the hypothalamus and pituitary functions, and causes the disorders of hormone secretion. At the same time, the reproductive organs were injured by Aroclor 1254; as a result, the embryo was damaged in the development process [19]. Humans and animals are daily exposed to chemical pollutants that could adversely influence physiological processes and potentially cause diseases, including endometriosis, inhibition of estrogen-induced increases, DNA synthesis, and gene-expression responses [20].

PCBs enhanced CYP450 activities in animals [21, 22]. By MTT and Western blot methods, our results proved that the expression of CYP450 increased gradually with the treatment of the gradient Aroclor 1254; the highest expression was observed under 1 *μ*g/mL Aroclor 1254 treatment. When the concentration of Aroclor 1254 reached 10 *μ*g/mL, the expression of CYP450 started to decline [23]. The expression of CYP450 was related closely to the release of cytokines; more cytokine release led to the inhibition of CYP450 enzyme functions [24]. Macrophage was one of the main immunocytes in mammal's intrauterine; it could produce nitric oxide (NO) and tumor necrosis factor (TNF-*α*); higher TNF-*α* could affect normal fetal growth seriously and cause embryonic loss or even abortion. IL-6 was the sensitive index in diagnosis of endometriosis [25]. In this experiment, the contents of TNF-*α* and IL-6 in the injured model increased significantly than that in the control group; it is suggested that Aroclor 1254 could induce endometrial cells inflammation. Aroclor 1254 affected cell viability, increased the proportion of necrotic cells [26], and reduced the activity of CYP450; all above results demonstrated that the Aroclor 1254 injured endometrial cells model was made successfully. In addition, E_2_ and P_4_ were the main hormones in maintaining normal animal pregnancy. Yoshizawa et al. [27] studies suggest that PCB153 possesses estrogenic properties and competes with estrogen at the estrogen receptor. In this experiment, Aroclor 1254 significantly increased the production of E_2_ and significantly reduced P_4_. It is indicated that Aroclor 1254 induced disorders of hormone secretion, the steroid hormone physiological functions, and had direct adverse effect on embryo development.

As one type of flavonoids, quercetin possesses multiple biological activities. Such as antioxidant activity, DNA damage protectors,and preventing free radical-mediated cytotoxicity [28]. Quercetin displayed protective effects on spermatogonial cells from A1254-induced oxidative damage through increasing intracellular antioxidant levels and decreasing lipid peroxidation. Lin et al. [29] claimed that quercetin had specific protective effect on the hepatocytes injured by lipopolysaccharide (LPS) and could reduce the level of TNF-*α*. In addition, quercetin had protective effect on blood vessel endothelium cells injured by TNF-*α*. Liu et al. [30] and Kempuraj et al. [31] also reported that quercetin could inhibit the secretion of IL-6 and exert anti-inflammatory actions. In this paper, with the treatment of quercetin, the contents of TNF-*α* and IL-6 declined significantly than those in Aroclor 1254 group, it indicated that quercetin inhibited secretion of TNF-*α* and IL-6. Jeong et al. [32] found that quercetin had strong cytoprotective effects on H2O2-induced cell death, the vacuoles in the endometrial cells disappeared, and the cells recovered to normal after quercetin treatment. These results indicated that quercetin had protective effect on the injured cells induced by Aroclor 1254. Quercetin had estrogen-like effects; low concentration of quercetin could increase the level of estradiol [33]. In this paper, the contents of E_2_ decreased and P_4_ increased significantly with quercetin treatment; the results were in accordance with related reports. Therefore, quercetin plays an important role in promoting pregnancy and preventing miscarriages.

As the important drug metabolism enzyme, CYP450 had many members. Among them, CYP1A1 participated mainly in detoxicating and activating process of exogenous substances; it was the important metabolic enzyme of some procarcinogens and toxins and participated in hydroxylation metabolism of estradiols [34]. Mutant alleles of the CYP 1A1 gene are major modulators of lung cancer risk among smokers, mediate gender differences in lung cancer susceptibility, and associate with an elevated risk for breast, prostate, colorectal, and oral squamous cell cancer [35]. CYP2B isoforms indicate that the PCBs induce an active hepatic metabolic state that might produce the biological character to potentially increase the risk of procarcinogen bioactivation in rats [36]. In this experiment, the injured rat endometrial cells were treated with various concentrations of quercetin, the results showed that the expression of CYP2B1 increased gradually with the gradient quercetin treatment, and the optimal expression was 50 *μ*mol/L quercetin treated for 24 h. The expression of CYP1A1 in the endometrial cells increased gradually with the treatment of gradient quercetin by RT-PCR, but the expression was not obvious by Western blot analysis. This result confirms the lack or low expression in rat fetuses of many CYP450 isoforms detected in adults, as reported by Czekaj et al. [37]. Nevertheless, whether CYP450 gene regulatory mechanisms are present in uterus tissues remains controversial.

All the results showed that the expression of CYP450 in the Aroclor 1254 injured rat endometrial cells was at its optimal level when they were treated with 50 *μ*mol/L quercetin. This indicates that 50 *μ*mol/L quercetin has the protective effect on injured endometrial cells of pregnant rats.

## 5. Conclusion

Quercetin has protective effects on the injured endometrial cells in the pregnant rats. This study can provide some useful information for herbal medicine protection from animal reproductive diseases caused by environmental endocrine disruptors.

## Figures and Tables

**Table 1:** The inhibitory effect of Aroclor 1254 on growth of normal endometrial cells (*n* = 6).

Aroclor 1254 (*μ*g/mL)	Absorbance value	IR (%)
0	0.435 ± 0.013^d^	0
0.01	0.440 ± 0.021^d^	—
0.1	0.432 ± 0.014^d^	0.69
1	0.416 ± 0.008^c^	4.37
10	0.382 ± 0.022^b^	12.18
100	0.234 ± 0.025^a^	46.21

Note: Cells viability was monitored by the MTT assay. Cells were treated with gradient Aroclor 1254 for 48 h, and the viability was monitored. Cell viability was expressed as mean ± SEM. The inhibition ratio (IR) = 1 − (the test group OD value/the control group OD value). Compared with the control group, values marked with different letters are significant (*P* < 0.05).

**Table 2:** The protective effect of quercetin on the injured endometrial cells for 24 h treatment (*n* = 6).

Quercetin (µmol/L)	24 h		48 h		72 h	
	Absorbance	Viability (%)	Absorbance	Viability (%)	Absorbance	Viability (%)
0	0.431 ± 0.003^f^	100	0.402 ± 0.005^e^	93.27	0.389 ± 0.006^e^	90.26
10 *μ*g/mL Aroclor 1254	0.346 ± 0.004^d^	80.28	0.255 ± 0.012^b^	59.16	0.186 ± 0.011^b^	43.16
10	0.379 ± 0.003^e^	87.94	0.289 ± 0.004^c^	67.05	0.245 ± 0.006^b^	56.84
50	0.401 ± 0.010^e^	93.04	0.318 ± 0.007^c^	73.78	0.251 ± 0.013^b^	58.24
100	0.325 ± 0.005^c^	75.41	0.264 ± 0.008^b^	61.25	0.202 ± 0.004^b^	46.87
500	0.057 ± 0.005^a^	12.23	0.037 ± 0.014^a^	8.58	0.021 ± 0.015^a^	4.87

Note: The viabilities of the normal cells, the injured cells, and the quercetin treated cells were monitored by the MTT assay. The optimal viability was obtained by 50 µmol/L quercetin treated for 24 h. Cell viability was expressed as mean ± SEM. Compared with the control group, values marked with different letters are significant (*P* < 0.05).

**Table 3:** Effect of quercetin on the contents of TNF-*α*, IL-6, E_2_, and P_4_ in the injured endometrial cells (*n* = 6).

	Control group	Aroclor 1254 group	50 µmol/L quercetin group
TNF-*α* (pg)	10.99 ± 1.12^a^	25.50 ± 2.52^c^	15.95 ± 1.61^b^
IL-6 (pg)	27.17 ± 2.95^a^	73.34 ± 12.93^b^	39.77 ± 6.54^a^
E_2_ (pg)	203.09 ± 11.37^a^	267.34 ± 12.24^b^	215.64 ± 20.12^a^
P_4_ (ng)	0.51 ± 0.028^a^	0.12 ± 0.037^b^	0.47 ± 0.156^a^

Note: The contents of TNF-*α*, IL-6, E_2_, and P_4_ in the control group, the injured endometrial cells model group, and the quercetin group were monitored by the ELISA. Data were expressed as treatment mean ± SEM (*n* = 6). Compared with the control group, values marked with different letters are significant (*P* < 0.05).

## References

[1] Ramadass P, Meerarani P, Toborek M, Robertson LW, Hennig B (2003). Dietary flavonoids modulate PCB-induced oxidative stress, CYP1A1 induction, and AhR-DNA binding activity in vascular endothelial cells. *Toxicological Sciences*.

[2] Wakui S, Takagi F, Muto T (2007). Spermatogenesis in aged rats after prenatal 3,3′,4,4′,5-pentachlorobiphenyl exposure. *Toxicology*.

[3] Lin P, Chang JT, Ko J-L, Liao S-H, Lo W-S (2004). Reduction of androgen receptor expression by benzo[a]pyrene and 7,8-dihydro-9,10-epoxy-7,8,9,10-tetrahydrobenzo[a]pyrene in human lung cells. *Biochemical Pharmacology*.

[4] Cohn BA, Terry MB, Plumb M, Cirillo PM (2012). Exposure to polychlorinated biphenyl (PCB) congeners measured shortly after giving birth and subsequent risk of maternal breast cancer before age 50. *Breast Cancer Research and Treatment*.

[5] Chen C-Y, Hamm JT, Hass JR, Birnbaum LS (2001). Disposition of polychlorinated dibenzo-p-dioxins, dibenzofurans, and non-ortho polychlorinated biphenyls in pregnant Long Evans rats and the transfer to offspring. *Toxicology and Applied Pharmacology*.

[6] Yang F, Xu Y (2005). Hydroxylated metabolites of polychlorinated biphenyls and their endocrine disrupting mechanism. *Progress in Chemistry*.

[7] Chu S, Covaci A, Schepens P (2003). Levels and chiral signatures of persistent organochlorine pollutants in human tissues from Belgium. *Environmental Research*.

[8] Kholkute SD, Rodriguez J, Dukelow WR (1994). Effects of polychlorinated biphenyls (PCBs) on in vitro fertilization in the mouse. *Reproductive Toxicology*.

[9] Crinnion WJ (2011). Polychlorinated biphenyls: persistent pollutants with immunological, neurological, and endocrinological consequences. *Alternative Medicine Review*.

[10] Fadhel Z, Lu Z, Robertson LW, Glauert HP (2002). Effect of 3,3′,4,4′-tetrachlorobiphenyl and 2,2′,4,4′,5,5′-hexachlorobiphenyl on the induction of hepatic lipid peroxidation and cytochrome P-450 associated enzyme activities in rats. *Toxicology*.

[11] Pawlikowska-Pawlȩga B, Gruszecki WI, Misiak LE, Gawron A (2003). The study of the quercetin action on human erythrocyte membranes. *Biochemical Pharmacology*.

[12] Egert S, Wolffram S, Bosy-Westphal A (2008). Daily quercetin supplementation dose-dependently increases plasma quercetin concentrations in healthy humans. *Journal of Nutrition*.

[13] Liu C-Y, Lin Y-C, Deng J-S, Liao J-C, Peng W-H, Huang G-J (2012). Antioxidant, anti-inflammatory, and antiproliferative activities of Taxillus sutchuenensis. *American Journal of Chinese Medicine*.

[14] Musonda CA, Chipman JK (1998). Quercetin inhibits hydrogen peroxide (H2O2)-induced NF-*κ*B DNA binding activity and DNA damage in HepG2 cells. *Carcinogenesis*.

[15] Bandera EV, Williams MG, Sima C (2009). Phytoestrogen consumption and endometrial cancer risk: a population-based case-control study in New Jersey. *Cancer Causes and Control*.

[16] Bovet C, Plet B, Ruff M (2009). Towards high-throughput identification of endocrine disrupting compounds with mass spectrometry. *Toxicology in Vitro*.

[17] Cui X, Thomas A, Han Y (2005). Quantitative PCR assay for cytochromes P450 2B and 3A induction in rat precision-cut liver slices: correlation study with induction *in vivo*. *Journal of Pharmacological and Toxicological Methods*.

[18] Martignoni M, de Kanter R, Grossi P, Mahnke A, Saturno G, Monshouwer M (2004). An *in vivo* and *in vitro* comparison of CYP induction in rat liver and intestine using slices and quantitative RT-PCR. *Chemico-Biological Interactions*.

[19] Hernández-Ochoa I, Karman BN, Flaws JA (2009). The role of the aryl hydrocarbon receptor in the female reproductive system. *Biochemical Pharmacology*.

[20] Bellelis P, Podgaec S, Abrão MS (2011). Environmental factors and endometriosis. *Revista da Associação Médica Brasileira*.

[21] Wiseman S, Vijayan MM (2011). Aroclor 1254 disrupts liver glycogen metabolism and enhances acute stressor-mediated glycogenolysis in rainbow trout. *Comparative Biochemistry and Physiology C*.

[22] Yuan J, Lu W-Q, Zou Y-L (2009). Influence of aroclor 1254 on benzo(a)pyrene-induced DNA breakage, oxidative DNA damage, and cytochrome P4501A activity in human hepatoma cell line. *Environmental Toxicology*.

[23] Zou Y-L, Lai R-P, Zhou L-H, Li X-Y, Lu W-Q (2006). Enhancement effect of polychlorinated biphenyl on benzo (a) pyrene-induced DNA damage in HepG2 cells. *Chinese Journal of Preventive Medicine*.

[24] Renton KW, Knickle LC (1990). Regulation of hepatic cytochrome P-450 during infectious disease. *Canadian Journal of Physiology and Pharmacology*.

[25] Li DJ, Liu YF, Pei XY, Guo DZ (2010). Research on change of acute phase protein and IL-6 in cows with endometritis. *Acta Veterinaria et Zootechnica Sinica*.

[26] Bredhult C, Bäcklin B-M, Olovsson M (2007). Effects of some endocrine disruptors on the proliferation and viability of human endometrial endothelial cells in vitro. *Reproductive Toxicology*.

[27] Yoshizawa K, Brix AE, Sells DM (2009). Reproductive lesions in female Harlan Sprague-Dawley rats following two-year oral treatment with dioxin and dioxin-like compounds. *Toxicologic Pathology*.

[28] Zhang YMC (2005). Protective effect of quercetin on aroclor 1254-induced oxidative damage in cultured chicken spermatogonial cells. *Toxicological Sciences*.

[29] Lin R, Liu J, Gan W (2004). Protection of vascular endothelial cells from TNF-alpha induced injury by quercetin. *Zhong Yao Cai*.

[30] Liu J, Li X, Yue Y, Li J, He T, He Y (2005). The inhibitory effect of quercetin on IL-6 production by LPS-stimulated neutrophils. *Cellular & Molecular Immunology*.

[31] Kempuraj D, Castellani ML, Petrarca C (2006). Inhibitory effect of quercetin on tryptase and interleukin-6 release, and histidine decarboxylase mRNA transcription by human mast cell-1 cell line. *Clinical and Experimental Medicine*.

[32] Jeong Y-M, Choi Y-G, Kim D-S (2005). Cytoprotective effect of green tea extract and quercetin against hydrogen peroxide-induced oxidative stress. *Archives of Pharmacal Research*.

[33] Ternaux JP, Portalier P (2002). Effect of quercetine on survival and morphological properties of cultured embryonic rat spinal motoneurones. *Neuroscience Letters*.

[34] Badawi AF, Cavalieri EL, Rogan EG (2001). Role of human cytochrome P450 1A1, 1A2, 1B1, and 3A4 in the 2-, 4-, and 16*α*-hydroxylation of 17*β*-estradiol. *Metabolism*.

[35] Huber JC, Schneeberger C, Tempfer CB (2002). Genetic modeling of estrogen metabolism as a risk factor of hormone-dependent disorders. *Maturitas*.

[36] Wakui S, Yokoo K, Takahashi H (2006). Prenatal 3,3′,4,4′,5-pentachlorobiphenyl exposure modulates induction of rat hepatic CYP 1A1, 1B1, and AhR by 7,12-dimethylbenz[a]anthracene. *Toxicology and Applied Pharmacology*.

[37] Czekaj P, Wiaderkiewicz A, Florek E, Wiaderkiewicz R (2005). Tobacco smoke-dependent changes in cytochrome P450 1A1, 1A2, and 2E1 protein expressions in fetuses, newborns, pregnant rats, and human placenta. *Archives of Toxicology*.

